# Irradiated Pollen-Induced Parthenogenesis for Doubled Haploid Production in Sunflowers (*Helianthus* spp.)

**DOI:** 10.3390/plants12132430

**Published:** 2023-06-23

**Authors:** Hongxia Wang, Hongyan Hou, Chao-Chien Jan, Wun S. Chao

**Affiliations:** 1 USDA-ARS, Edward T. Schafer Agricultural Research Center, Fargo, ND 58102, USA; hongxia.wng@gmail.com (H.W.); chaochien.jan@gmail.com (C.-C.J.); 2 Mathematics Department, Minnesota State University, Moorhead, MN 56563, USA; hongyan388@gmail.com

**Keywords:** doubled haploid technology, gamma-induced parthenogenesis, sunflower (*Helianthus* spp.)

## Abstract

Doubled haploid (DH) technology is a tool used to develop large numbers of inbred lines and increase the rate of genetic gain by shortening the breeding cycles. However, previous attempts to produce DH sunflower plants (*Helianthus* spp.) have resulted in limited success. In this research, we applied gamma-induced parthenogenesis to assist the production of DH sunflowers. The objectives of the study included (1) identifying optimal gamma ray doses for inducing DH sunflowers using two cytoplasmic male sterility (CMS) lines as female plants and two male pollinators with recognizable morphological markers, (2) selecting new male pollinators from wild sunflower varieties, and (3) testing the efficacy of the selected male pollinators using emasculated non-male sterile sunflower lines as female plants. In these experiments, pollen grains were irradiated with gamma ray doses ranging from 50 to 200 Gy. The optimal gamma ray dose for pollen grain irradiation and DH plant production was identified to be 100 Gy. In addition, a cultivated (G11/1440) and a wild-type (ANN1811) sunflower line can be used as common male pollinators for their distinctive morphological markers and wide capacity for DH induction by gamma-irradiated pollen grains.

## 1. Introduction

Because F_1_ progeny exhibit phenotypic superiority over their parents with regard to stature, biomass, and fertility—a phenomenon referred to as “hybrid vigor” or “heterosis” [1]—the production of F_1_ hybrid seeds is important for farmers and breeders. In the development of F_1_ hybrids, the production of inbred parental lines is the first prerequisite and one of the most arduous steps, taking 6–10 generations of selfing or sibling crossing to obtain homozygous inbred lines [2]. Doubled haploid (DH) technology is a tool used to increase the rate of developing inbred lines and genetic gain by shortening breeding cycles. Doubled haploid technology relies on the development of haploid embryos. Once the haploid embryo is obtained, it may grow into a diploid voluntarily, or other treatments may be required for chromosome doubling [3,4].

In addition to the F_1_ heterosis mentioned above, haploid and DH plants have applications in many areas, such as genetic mapping, gene cloning, and breeding for genetic studies (reviewed in [5]). Haploid plants can be generated by in vitro (procedures are performed outside a plant) and in vivo (procedures are applied in plant) systems. The in vitro system including anther culture, isolated microspore culture, and in vitro-induced parthenogenesis (an unfertilized egg developing into an embryo) is based on the culture of immature male or female gametophytic cells [6,7]. The in vivo system includes chromosome elimination and irradiated pollen-induced parthenogenesis. Chromosome elimination is obtained from the progeny of inter- and intra-specific hybridization or targeted manipulation of the centromere-specific histone 3 variant (CENH3) [6,7,8]. Nonlethal gamma-irradiated pollen has been used to induce parthenogenesis in vivo from many species such as apple, cacao, cucumber, watermelon, muskmelon, and so forth [9,10,11,12,13], likely inciting the development of parthenogenetic diploid embryoids from egg cells. However, this method requires immature embryo rescue under in vitro conditions. In general, haploid induction rates for in vivo systems are influenced by the efficiency of haploid inducer lines [7].

In sunflowers, several methods, such as anther culture, microspore culture, and irradiation of pollen grains, have been evaluated by researchers to produce DH sunflower plants. However, previous attempts to produce DH plants have had limited success [14]. Although anther culture is one of the most used methods, cultivated sunflowers have proven to be very recalcitrant in anther culture, especially for shoot regeneration. Studies on the production of haploids via isolated microspores date back 30 years [15,16,17]; however, no full-grown haploid plants have been obtained using this method. The production of haploid sunflower plants via gamma-induced parthenogenesis has been attempted over the years with some success in obtaining haploid and DH plants [18,19,20,21]. However, due to many special requirements, such as the dependance of genotypes and the demand of specific doses of gamma rays for different genotypes, this method has not been practically applied.

In this research, we employed irradiated pollen-induced parthenogenesis for DH production in the sunflowers. The major objectives of this study were to develop an efficient and easy-to-implement protocol for DH production and to identify effective male pollinators. To simplify the experimental procedure for DH identification, cytoplasmic male sterility (CMS) lines were used as female parents, and sunflower lines homozygous for purple hypocotyl color were used as male pollinators. The optimal gamma ray doses and male pollinators were determined based on the efficiency of DH production.

## 2. Results and Discussion

### 2.1. Progeny Evaluation

**Figure 1:**
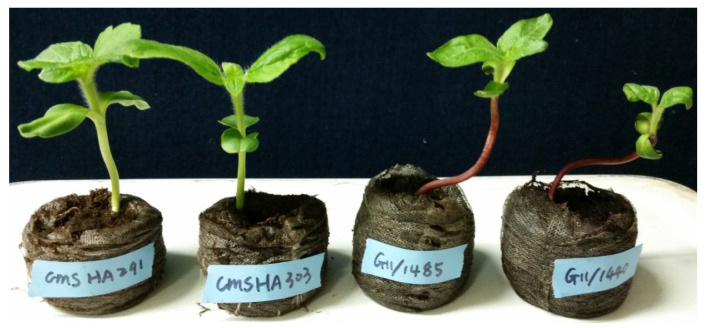
Contrasting hypocotyl colors of four parental seedlings, two female cytoplasmic male sterility (CMS) lines (CMS HA 291 and CMS HA 303) and two cultivated male pollinators (G11/1440 and G11/1485).

**Figure 2:**
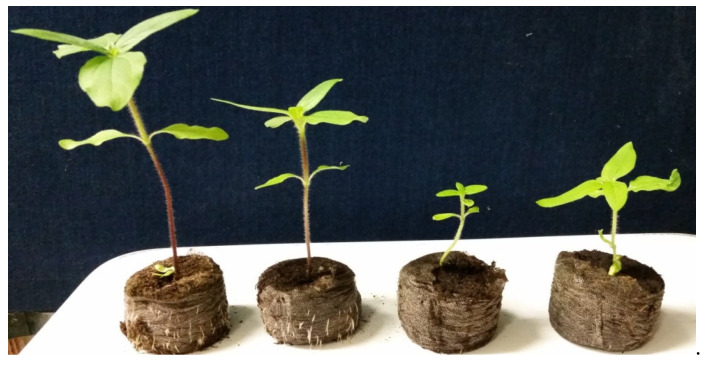
Contrasting hypocotyl color (purple and green) of offspring seedlings.

**Figure 3:**
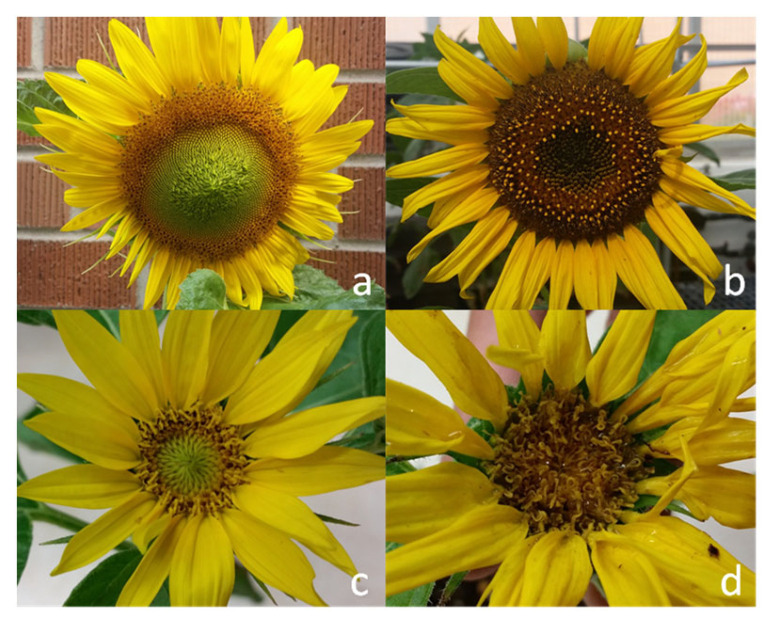
Flowers of parents and offspring. (**a**) CMS HA 303; (**b**) G11/1440; (**c**,**d**) yellow sterile flowers of offspring individuals with green hypocotyls.

To identify optimal gamma ray doses for the production of DH plants, two cytoplasmic male sterility (CMS) lines, CMS HA 291 and CMS HA 303, were used as female parents (Figure 1). These two CMS lines have green hypocotyls and maintain female fertility but do not produce viable pollen grains. In addition, two cultivated lines, G11/1440 and G11/1485 (Figure 1), that are homozygous for purple hypocotyl color were used as male pollinators; the hybrid seeds obtained from the cross between the pollinator (G11/1440 or G11/1485) and CMS (CMS HA 291 or CMS HA 303) lines produce purple hypocotyl seedlings (Figure 2). Any offspring seedlings with green hypocotyl color were potential DH/haploid plants and were grown continually for further progeny evaluation, which included flower color, fertility, and molecular marker testing (Supplemental Tables S1 and S2), whereas seedlings with purple hypocotyls were hybrid and discarded. Flower color and fertility were evaluated at the full blooming stage; the potential DH/haploid offspring were infertile and produced yellow flowers like their female parents, CMS HA 291 and CMS HA 303 (Figure 3).

Simple sequence repeat (SSR) markers were applied for molecular marker testing since many sunflower SSR markers were developed in the past and assigned to specific chromosomes (http://compositdb.ucdavis.edu, accessed on 15 May 2022). SSRs consist of short tandem repeats of 1–6 nucleotide sequences; they are codominant markers and are generally abundant and polymorphic due to their high mutation rates [22]. Eighty-five pairs of the developed SSR primers (see Supplemental Table S2, spreadsheet “SSR primers”) were chosen and tested using PCR to examine polymorphisms in four parental pairs (CMS HA 291 and G11/1440; CMS HA 303 and G11/1440; CMS HA 291 and G11/1485; CMS HA 303 and G11/1485). Our assessment showed that there were one to four polymorphic SSR markers in each of the four parental pairs for all 17 chromosomes (Supplemental Table S2, spreadsheet “number of polymorphic SSRs”). The results also indicate that 40% to 48% of the 85 SSR primer pairs showed polymorphism in those four parental pairs. For progeny evaluation, one to two SSR markers per chromosome that showed polymorphism in all four parental pairs were used to verify potential DH lines in offspring individuals with green hypocotyls (Supplemental Table S2, spreadsheet “SSR markers for CMS crosses”). A representative gel image and genotype data are shown in Supplemental Figure S1.

Besides morphological markers (hypocotyl color, flower color, and fertility) and molecular markers (SSRs) as mentioned above, chromosome number was also determined from the embryo-rescued sunflower plants. Root tips were collected from individuals with green hypocotyl and stained for chromosome counting. Because some of the offspring individuals were dead or too weak to collect suitable root tips, chromosome number was identified only for those that had healthy roots. Our results show that the majority of parthenogenetic offspring had 34 chromosomes (Supplemental Table S1, see column heading “Offspring chromosome #” in spreadsheet “CMS crosses”). These results indicate a very high frequency of spontaneous diploidization, which is consistent with the study by Todorova et al. [18]. In addition, any offspring individuals that had yellow and sterile flowers were verified to be DH lines (Supplemental Table S1, spreadsheet “CMS crosses”). We also detected abnormal chromosome number (aneuploidy) of 2n = 31, 32 or 33 in both DH and hybrid lines, a few haploid individuals with a chromosome number of 2n = 17 in parthenogenetic lines, and mixoploids (chimeras) that had 17 or 34 chromosomes per cell in parthenogenetic lines (a representative chromosome counting image is shown in Supplemental Figure S2). Spontaneous DHs and mixoploids took place when endomitosis or nuclear fusion occurred [23]; however, in spontaneous DHs, endomitosis or nuclear fusion might occur at the early stages of egg cell development, whereas in mixoploids, the chromosome-doubling process might occur later in the development of egg cell. Chromosome counting is thus essential for the detection of normal DH plants.

### 2.2. Identification of Optimal Gamma Ray Doses with Higher Efficiency and Frequency in Inducing Parthenogenesis

Pollen irradiation for parthenogenesis involves the pollination of female florets with gamma-irradiated pollen grains, followed by embryo rescue from the ovules. Preliminary tests indicated that gamma ray doses could be as high as 200 Gy for obtaining viable embryos if freshly collected pollen grains were irradiated with gamma rays and applied onto female sunflower heads immediately after irradiation. To identify optimal gamma ray doses, we examined the efficacy of DH production using a range of gamma ray doses, including 50, 100, 130, 150, 170, and 200 Gy, in four separate experiments (crossing dates). The effects of pollen irradiation on embryo growth and parthenogenetic plant production of the four crosses (CMS HA 291 × G11/1440, CMS HA 291 × G11/1485, CMS HA 303 × G11/1440, and CMS HA 303 × G11/1485) are recorded in Supplemental Table S1 (spreadsheet “CMS crosses”). Table 1 only presents the results when at least one DH or haploid offspring was obtained from any of the four crosses using the six different gamma ray doses mentioned above (see also Supplemental Table S1, spreadsheet “CMS progeny evaluation”).

**Figure 4:**
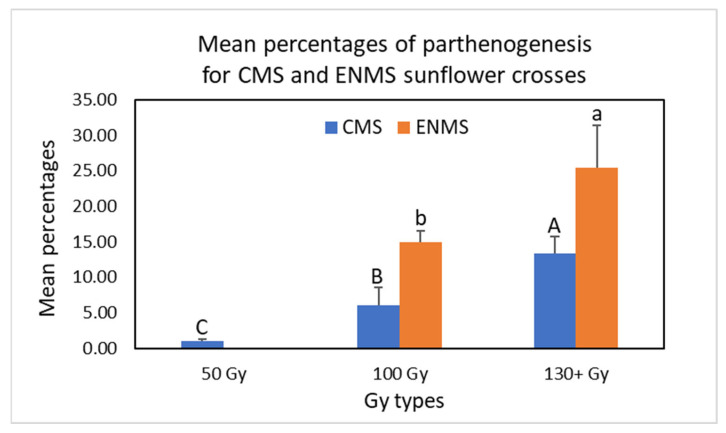
The mean percentages of parthenogenesis of different gamma ray dose (Gy) types. The Kruskal–Wallis test was used to analyze the equality of the mean percentages of parthenogenesis for cytoplasmic male sterility (CMS) sunflower crosses; in this analysis, 130+ Gy is the combined data of 130 Gy, 150 Gy, 170 Gy, and 200 Gy. The Wilcoxon two-sample test was used to analyze the equality of the mean percentages of parthenogenesis for emasculated non-male sterile (ENMS) sunflower crosses; in this analysis, 130+ Gy is the combined data of 130 Gy, 150 Gy, and 200 Gy. Error bars show range of the mean percentages of parthenogenesis. Mean percentages labeled with different letters are significantly dissimilar (*p* < 0.05).

**Figure 5:**
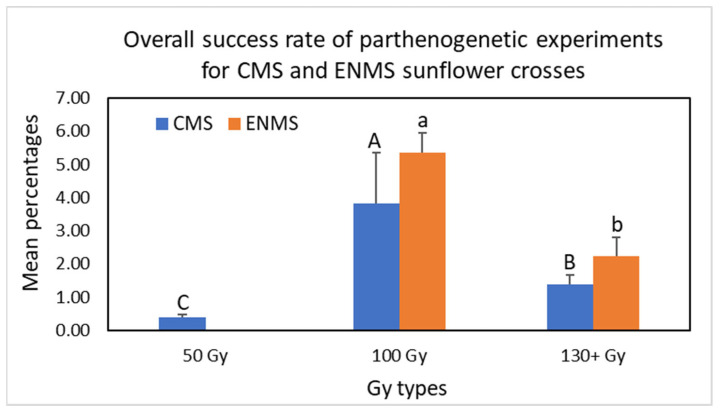
Overall success rate of parthenogenetic experiments for CMS and ENMS sunflower crosses. The Kruskal–Wallis test was used to analyze the overall success rate of parthenogenetic experiments for cytoplasmic male sterility (CMS) sunflower crosses; in this analysis, 130+ Gy is the combined data of 130 Gy, 150 Gy, 170 Gy, and 200 Gy. The Wilcoxon two-sample test was used to analyze the overall success rate of parthenogenetic experiments for emasculated non-male sterile (ENMS) sunflower crosses; in this analysis, 130+ Gy is the combined data of 130 Gy, 150 Gy, and 200 Gy. Error bars show range of the mean percentages of parthenogenesis. Mean percentages labeled with different letters are significantly dissimilar (*p* < 0.05).

The total numbers of rescued embryos and survived plants were greater for pollen grains irradiated with a gamma ray dose of 50 Gy compared to those irradiated with gamma ray doses above 50 Gy (Table 1 and Supplemental Table S1, spreadsheet “CMS crosses”); however, higher gamma ray doses resulted in a greater percentage of DH offspring production. For example, the percentage of parthenogenesis was 0.27–1.82% when pollen grains were irradiated with 50 Gy, whereas 1.00–25%, 3.85–12.5%, 20%, 11.11%, and 14.29–18.18% of DH offspring were obtained using pollen grains irradiated with 100, 130, 150, 170, and 200 Gy, respectively, among all the DH plants obtained from four crosses (Table 1). Statistical analysis of the mean percentages of parthenogenesis indicated that three Gy groups, 50 Gy, 100 Gy, and 130+ Gy (130 Gy, 150 Gy, 170 Gy, and 200 Gy), were significantly different (*p* = 0.0031) in DH offspring production. The greater the gamma ray dose, the greater the mean percentage of parthenogenesis obtained (Figure 4 and Supplemental Table S3); the mean percentage of parthenogenesis for 50 Gy, 100 Gy, and 130+ Gy were 1.04%, 6.06%, and 13.32%, respectively. Although increased levels of gamma ray doses (i.e., 130+ Gy) appeared to be more efficient for DH offspring production, the number of DH plants obtained was low, not to mention that many higher gamma ray doses resulted in zero DH plant production in these four crosses. Statistical analysis showed that 100 Gy had the highest frequency of success of the parthenogenetic experiments (3.12%, *p* = 0.0028) compared to 50 Gy (0.4%) and 130+ Gy (1.37%) (Figure 5) after multiplying a coefficient of the overall success rates of the experiments (see Supplemental Table S3 for detailed data analysis). Therefore, based on our collective results, the 100 Gy gamma ray dose appears to be optimal for sunflower DH plant production, and this gamma ray dose is recommended when testing new male pollinators for DH plant production. Mysteriously, the 100 Gy gamma ray dose is much lower than previously reported gamma ray doses [18,19]. These authors tested gamma-ray-induced parthenogenesis in sunflowers using 300 Gy, 600 Gy, and 900 Gy, and the highest efficiency was achieved when pollination was carried out with pollen irradiated at 600 Gy. This discrepancy may be due to the different gamma ray instruments and diverse sunflower genotypes used in those experiments.

### 2.3. Selection of Additional Male Pollinator Lines

To identify alternative male pollinators, wild-type sunflower pollen was tested to determine if parthenogenesis could be induced without the need for gamma ray irradiation, as has been implemented in maize [24,25]. Twenty-four wild sunflower accessions were selected (Supplemental Table S4), and the rate of seed germination and pollination were assessed (Supplemental Table S4) to determine which of these wild sunflower accessions were simple to grow and maintain. Among the 24 wild sunflower accessions treated with gibberellic acid (0.1 g/L), 12 had germination rates above 50% and 5 had germination rates of 10% to 50%. To evaluate the pollination rate (the number of seeds divided by the total number of flowers) from wide crosses between wild accessions and female CMS lines (CMS HA 291 and CMS HA 303), freshly collected pollen grains (without irradiation) were applied onto sunflower heads. The results show that the pollination rates of annual accessions could be as high as 72%; however, most perennial species had very low pollination rates (0–3%). Annual accession ANN1811 had the best pollination rate after being crossed with CMS HA 291 and CMS HA 303 (67% and 72%, respectively). Another annual accession, PET 478, had a 52% pollination rate after being crossed with CMS HA 291, but no fertilization took place after it was crossed with CMS HA 303. Three other annual accessions (PRA416, 33, and PRA434) had substantially lower pollination rates (10% to 12%) after being crossed with CMS HA 291. Wild accessions that had a reasonable germination rate (≥30%) and pollination rate (≥10%) were used as pollen sources to induce parthenogenesis.

Efficiency of DH induction was evaluated using irradiated and non-irradiated pollen grains. Most offspring individuals had branches (a paternal trait) and pollen when non-irradiated pollen grains were used to pollinate female heads, and all of these offspring individuals were confirmed to be hybrids, indicating that non-irradiated pollen grains of wild sunflower species may be incapable of inducing DH offspring. When using irradiated pollen grains, only ANN1811 exhibited embryo production and DH induction; six plants survived after embryo rescue from CMS HA 291 flowerhead pollinated with ANN1811 pollen irradiated with 100 Gy, and one of them was verified to be a DH plant (Table 2). This result indicates that ANN1811 had the potential for parthenogenesis induction and was thus used as an additional pollen source for DH production in emasculated non-male sterile (ENMS) cultivated sunflower lines, as mentioned below.

### 2.4. DH Plant Production Using Emasculated Non-Male Sterile (ENMS) Cultivated Sunflower Lines

For practical use of parthenogenesis technology, we examined the production of DH plants using ENMS cultivated sunflower lines. In this experiment, three cultivated sunflower lines, HA 60, HA 89, and HA 232 were used as female plants, and G11/1440, G11/1485, and ANN1811 were used as male pollinators. To induce male sterility in female sunflower heads, 125 ppm gibberellic acid (GA) was applied to the floral heads (HA 60, HA 89, and HA 232) at the R2 stage, which thoroughly prevented pollen production in those floral heads (see Supplemental Figure S3 for evaluation of effective GA concentrations). The effectiveness of DH production was examined using pollen grains irradiated with 100, 130, 150, 170, and 200 Gy in two separate experiments (crossing dates). The impacts of pollen irradiation on embryo rescue and parthenogenetic plant production of the nine crosses (HA 60 × G11/1440, HA 60 × G11/1485, HA 60 × ANN1811, HA 89 × G11/1440, HA 89 × G11/1485, HA 89 × ANN1811, HA 232 × G11/1440, HA 232 × G11/1485, and HA 232 × ANN1811) are recorded in Supplemental Table S1 (spreadsheet “ENMS crosses”). Table 2 presents the results when at least one DH or haploid offspring was obtained from any of the nine crosses using the five different gamma ray doses mentioned above (see also Supplemental Table S1, spreadsheet “ENMS progeny evaluation”).

Similar to the aforementioned experiments, increased levels of gamma ray doses resulted in a greater percentage of parthenogenesis; however, no DH plants were obtained using 170 Gy. The percentages of DH offspring for 100, 130, 150, and 200 Gy were 6.25–20%, 8.33%, 22.22–26.31, and 26–45.46%, respectively, among all the DH plants obtained from nine crosses (Table 2). Statistical analysis of the mean percentages of parthenogenesis indicated that 100 Gy and 130+ Gy (130 Gy, 150 Gy, and 200 Gy) had significantly different (α = 0.05) impacts on the success rate of parthenogenetic plant production (Figure 4 and Supplemental Table S3). Again, increased levels of gamma ray doses resulted in a greater mean percentage of parthenogenesis; the mean percentage for 100 Gy and 130+ Gy was 14.88% and 25.47%, respectively. However, although increased gamma ray doses (i.e., 130+ Gy) appeared to be more efficient for DH offspring production, 100 Gy led to a higher overall success rate of parthenogenetic experiments (5.36%, α = 0.05) compared to 130+ Gy (2.24%) among the nine crosses (Figure 5 and Supplemental Table S3). Therefore, the 100 Gy gamma ray dose still performed best for sunflower DH plant production using ENMS sunflower lines as female plants. The results also indicate that the female plant HA 232 generated fewer embryos than HA 60 and HA 89, and no DH plants were obtained from any of its three aforementioned crosses. In addition, no DH plants were obtained using G11/1485 as a male pollinator. Varying parthenogenetic responsiveness among different female and male genotypes was also observed by other investigators [19,21,26]. Table 3 provides all DH offspring obtained from plant materials used in this project. Our results show that two sunflower lines (G11/1440 and ANN1811) can be used as common male pollinators for induction of DH in desired sunflower lines using gamma-irradiated pollen grains, and the recommended gamma ray dose for pollen grain irradiation is 100 Gy, even though a greater dose may generate a higher percentage of DH sunflowers.

## 3. Materials and Methods

### 3.1. Plant Materials

To prevent self-pollination, two cytoplasmic male sterility (CMS) lines, CMS HA 291 (PI 650607; pedigree INRA 6501 selfing) and CMS HA 303 (PI 650611; pedigree Voshod PI 371936 selfing), were used as female parents, and they were kept at the USDA Sunflower and Plant Biology Research Unit (SPBRU) in Fargo, ND, USA. Two cultivated lines, G11/1440 (HA 434 × PH3, F_2_) and G11/1485 (HA 434 × PH3, F_2_) were used as male pollinators. Both G11/1440 and G11/1485 were developed by Dr. C.C. Jan and kept at the SPBRU. These two lines were selected from the F_2_ population of the cross between HA 434 and PH3 and were confirmed to be homozygous for purple hypocotyl color and purple stem color (about 5 cm in length) at the base of the plant. A total of 24 wild *Helianthus* accessions (Supplemental Table S4) were examined for the identification of alternative male pollinators, of which 22 were obtained from the U.S. National Plant Germplasm System (https://www.ars-grin.gov/npgs/, accessed on 15 May 2022), and the other 2 (NUT-102 and N-S-622) were developed at the SPBRU. Wild sunflower collection included three annual species (*H. annuus*, *H. petiolaris*, *H. praecox*) and two perennial species (*H. maximiliani* and *H. nuttallii*). HA 60 (pedigree 953-102-1-1-22/2*VNIIMK1646), HA 89 (pedigree VNIIMK 8931 Sel), and HA232 (pedigree 2*Smena//HA6/HA8) were used for emasculated non-male sterile (ENMS) crosses; they were maintainer sunflower lines and kept at the North Dakota Agricultural Experiment Station in Fargo, ND, USA.

### 3.2. Pollen Irradiation, Pollination, Embryo Rescue, and Culture Media

Fresh pollen samples collected from G11/1440 and G11/1485 and other genotypes were irradiated with a range of gamma ray doses, including 50, 100, 130, 150, 170, and 200 Gy. The source of gamma radiation was ^137^Cs. CMS or emasculated female floral heads were then hand-pollinated with irradiated pollen grains immediately. After growth for 6 days, the number of embryos at different developmental stages (fully developed, heart, early heart, and globular stages) were determined. In addition, immature embryos were rescued and cultured following the two-step procedure described by Sukno et al. [27]. Briefly, the immature embryos were first cultured on Gamborg B5 Medium containing all the necessary micronutrients and vitamins. After one to two weeks, the enlarged embryos were transferred to the germination medium with B5 salts, 10 g/L sucrose, and agar. Once seedlings were grown with well-developed root systems, the plantlets were transferred to Jiffy-7, placed in a growth chamber at 23–25 °C for one week, transplanted to clay pots, and subjected to progeny evaluation.

### 3.3. Progeny Evaluation Using Morphological and Molecular Markers

Progeny evaluation includes the use of morphological and molecular markers. Hypocotyl color, flower color, and fertility were considered as morphological markers for progeny evaluation. The flowers of the two male pollinators (G11/1440 and G11/1485) are purple and male-fertile, while the flowers of the female CMS lines (CMS HA 291 and CMS HA 303) are yellow and male-sterile. Since the two male pollinators possess the restoration of male fertility gene(s), the flowers of hybrid offspring were purple and male-fertile; however, the flowers of parthenogenetic offspring were yellow and male-sterile. The wild sunflower pollinator (ANN1811) is branched, and its flowers are purple and fertile.

Simple sequence repeats (SSRs) were used as molecular markers for DH confirmation. Eighty-five pairs of SSR primers mapped to the 17 sunflower linkage groups from the Compositae database (http://compositdb.ucdavis.edu, accessed on 15 May 2022) were used to screen polymorphism between male and female parents (for primer sequences, see Supplemental Table S2, spreadsheet “SSR primers”). PCR was performed using genomic DNA from leaf samples of parental lines, hybrids, and offspring with green hypocotyls. Extraction of genomic DNA was performed using a kit (Qiagen DNAeasy 96 Plant Kit, Qiagen, Valencia, CA, USA) following the manufacturer’s protocol. The concentration of DNA was quantified with a Nanodrop ND-1000 v3.5.2 spectrophotometer (Nanodrop Technology^®^, Cambridge, UK). The PCR amplification system was conducted following Liu et al. [28] with minor modifications. An amount of 15 μL of PCR reaction mixture contained 1× PCR buffer, 2 mM MgCl_2_, 0.2 mM dNTPs, 0.02 μM of the forward primer with an M13 tail (CACGACGTTGTAAAACGAC) at the 5′ end, 0.27 μM of the reverse primer, 0.27 μM of fluorescently labeled M13 primer, 40 ng of DNA, and 1 U Taq DNA polymerase (Qiagen). PCR amplifications were performed followed the “touchdown” procedures described by Qi et al. [29]. The initial denaturation step was performed at 95 °C for 3 min, followed by 10 cycles in which the annealing temperature was decreased by 0.5 °C for each cycle starting at 94 °C for 45 s, and then 64 °C for 45 s, and 72 °C for 1 min. An additional 35 cycles at 94 °C for 45 s, 58 °C for 45 s, and 72 °C for 1 min were performed thereafter. The PCR was completed with an extension step at 72 °C for 20 min. PCR product evaluation was carried out through gel electrophoresis using an IR2 4300/4200 DNA Analyzer (Li-COR, Lincoln, NE, USA) on 6.5% polyacrylamide gel.

### 3.4. Chromosome Counting

Chromosome numbers of the embryo-rescued sunflower plants were determined using Feulgen staining technique according to Jan [30]. Root tips were collected and placed in ice water for 18 h at 4 °C. The treated root tips were then fixed in 3:1 100% ethanol–glacial acetic acid solution for 4 h at room temperature, followed by hydrolyzing in 1N HCl at 60 °C for 12 min, and stained in Feulgen solution for 50 min at room temperature. The root tips were rinsed with tap water at least three times to remove staining solution until no color was shown in water. Enzymolysis was performed with 0.2% pectinase and 0.2% cellulase for 20 min at room temperature. The treated root tips were stored at 4 °C and examined using a Zeiss Axioplan 2 imaging microscope.

### 3.5. Emasculation for the Generation of Male-Sterile Female Parent

Gibberellic acid (GA) was used as a male sterilant. GA solution (125 ppm) was applied by spraying intact floral buds at the R2 stage. The terminal bud of sunflower at the R2 stage forms an immature bud 0.5 to 2.0 cm in size (see Supplemental Figure S3). GA solutions were sprayed at the center of the growing point. To make sure that no self-pollination occurred, flower heads were examined daily to verify that no pollen could be observed; any sunflower plants that showed pollen growth were discarded immediately.

### 3.6. Statistical Analysis

- Mean percentages of parthenogenesis for CMS and ENMS sunflower crosses (Figure 4).
  1. Analysis of the mean percentages of parthenogenesis for CMS sunflower crosses: The Kruskal–Wallis test was used to compare the differences among different Gy irradiation groups (for a detailed analysis, see Supplemental Table S3). Using CMS sunflower lines as female parents, results were obtained from 6 Gy irradiation groups (50 Gy, 100 Gy, 130 Gy, 150 Gy, 170 Gy, and 200 Gy). Because the sample sizes for 130 Gy, 150 Gy, 170 Gy, and 200 Gy were all below 5, and the Kruskal–Wallis test performs poorly, we combined the data of 130 Gy, 150 Gy, 170 Gy, and 200 Gy into one group and designated this group as 130+ Gy. In this analysis, $\mu_{1}$ is the mean percentage of parthenogenesis of 50 Gy, $\mu_{2}$ is the mean percentage of parthenogenesis of 100 Gy, and $\mu_{3}$ is the mean percentage of parthenogenesis of 130+ Gy. Other parameters include the following: k = the population number ($k=3),\;n_{i}$ = the size of sample i ($n_{1}$= 6, $n_{2}$=10, $n_{3}=6)$, $n_{T}\;$= total number of observations in all samples ($n_{T}=6+10+6=22),\;\mathrm{and}\;R_{i}=$ the sum of the ranks for sample i ($R_{1}=28,\;R_{2}=121,\;R_{3}=104$). The test statistics are as follows:
    $$H=\left[\frac{12}{n_{T}\left(n_{T}+1\right)}\;\sum_{i=1}^{k}\frac{R_{i}^{2}}{n_{i}}\;\right]-3\left(n_{T}+1\right)=\left[\frac{12}{22\left(22+1\right)}\;\left(\frac{28^{2}}{6}+\frac{121^{2}}{10}+\frac{104^{2}}{6}\right)\;\right]-3\left(22+1\right)=11.572.\;$$
    The test hypothesis H_o_ implies that the three mean percentages $\left(\mu_{1},\;\mu_{2},\;\mathrm{and}\;\mu_{3}\right)$ were all equal $\left(\mu_{1}=\mu_{2}=\mu_{3}\right)$, whereas $H_{1}$ implies that the three mean percentages were significantly different. The test statistics follow a $\chi^{2}$ distribution with 2 degrees of freedom, and the p value of the test is $p(\chi^{2}>11.572)$ = 0.0031, which is less than the chosen significant value of 0.05, indicating that the mean percentages of parthenogenesis for three Gy types (50 Gy, 100 Gy, and 130+ Gy) were significantly different.
  2. Analysis of the mean percentages of parthenogenesis for ENMS sunflower crosses: For studies using ENMS sunflower lines as female parents (including one result obtained from CMS 291 × ANN1811), results were obtained for four Gy irradiation groups (100 Gy, 130 Gy, 150 Gy, and 200 Gy). Since the sample size for the 130 Gy, 150 Gy, and 200 Gy groups was small, the data of these three Gy types were combined into one group, designated as 130+ Gy. The Wilcoxon two-sample test was used to determine if the mean percentage of parthenogenesis of 130+ Gy was higher than that of 100 Gy (for a detailed analysis, see Supplemental Table S3). In this analysis, $\mu_{1}$ is the mean percentage of parthenogenesis of 130+ Gy, $\mu_{2}$ is the mean percentage of parthenogenesis of 100 Gy, $n_{1}$ represents the sample size of 130+ Gy, $n_{2}$ represents the sample size of 100 Gy, $w_{1}$ represents the rank summation of 130+ Gy, $w_{2}$ represents the rank summation of 100 Gy, and the test hypotheses are $H_{0}:\mu_{1}=\mu_{2}$ and $H_{1}:\mu_{1}>\mu_{2}$. The test statistics are the minimum of $\mu_{1}$ and $\mu_{2},$ where $u_{1}=w_{1}-\frac{n_{1}\left(n_{1}+1\right)}{2}=48-\frac{5*6}{2}=33$, and $u_{2}=w_{2}-\frac{n_{2}\left(n_{2}+1\right)}{2}=43-\frac{8*9}{2}\;$= 7. The significance value α was designated as 0.05. According to the table of the Wilcoxon rank-sum test, the critical region was 8. Since $\mu_{2}=7,$ which is less than 8, the null hypothesis was rejected, and the analysis indicated that the mean percentage of parthenogenesis for 130+ Gy was significantly greater than that of 100 Gy.
- Overall success rates of parthenogenetic experiments for CMS and ENMS sunflower lines (Figure 5).
  1. Analysis of the overall success rates of parthenogenetic experiments for CMS sunflower crosses: To analyze the overall success rates of parthenogenetic experiments, the percentages of parthenogenesis were modified by multiplying a coefficient of the overall success rates of the experiments, and the Kruskal–Wallis test was used to analyze the equality of means (modified mean percentages of parthenogenesis) (for detailed a analysis, see Supplemental Table S3). In this analysis, $\mu_{1}$ is the mean percentage of parthenogenesis of 50 Gy, $\mu_{2}$ is the mean percentage of parthenogenesis of 100 Gy, and $\mu_{3}$ is the mean percentage of parthenogenesis of 130+ Gy. Other parameters include the following: k = the population number ($k=3),\;n_{i}$ = the size of sample i ($n_{1}$= 6, $n_{2}$=10, $n_{3}=6)$, $n_{T}\;$= the total number of observations in all samples ($n_{T}=6+10+6=22),\;\mathrm{and}\;R_{i}=$ the sum of the ranks for sample i ($R_{1}=24,\;R_{2}=154,\;R_{3}=75$). The test statistics are
    $$H=\left[\frac{12}{n_{T}\left(n_{T}+1\right)}\;\sum_{i=1}^{k}\frac{R_{i}^{2}}{n_{i}}\;\right]-3\left(n_{T}+1\right)=\left[\frac{12}{22\left(22+1\right)}\;\left(\frac{24^{2}}{6}+\frac{154^{2}}{10}+\frac{75^{2}}{6}\right)\;\right]-3\left(22+1\right)=11.75.$$
    The test hypothesis H_o_ implies that the three mean percentages $\left(\mu_{1},\;\mu_{2},\;\mathrm{and}\;\mu_{3}\right)$ were all equal $\left(\mu_{1}=\mu_{2}=\mu_{3}\right)$, whereas $H_{1}$ implies that the three mean percentages were significantly different. The test statistics follow a $\chi^{2}$ distribution with 2 degrees of freedom, and the p value of the test is $p(\chi^{2}>11.75)$ = 0.0028, which is less than the chosen significant value of 0.05, indicating that the success rates of parthenogenetic experiments for the three Gy types (50 Gy, 100 Gy, and 130+ Gy) were significantly different.
  2. Analysis of the overall success rate of parthenogenetic experiments for ENMS sunflower crosses: To analyze the overall success rates of parthenogenetic experiments, the percentages of parthenogenesis were modified by multiplying a coefficient of the overall success rates of the experiments, and the Wilcoxon two-sample test was used to determine if the modified mean percentage of parthenogenesis of 130+ Gy is smaller than that of 100 Gy (for a detailed analysis, see Supplemental Table S3). In this analysis, $\mu_{1}$ is the mean percentage of parthenogenesis of 130+ Gy, $\mu_{2}$ is the mean percentage of parthenogenesis of 100 Gy, $n_{1}$ represents the sample size of 130+ Gy, $n_{2}$ represents the sample size of 100 Gy, $w_{1}$ represents the rank summation of 130+ Gy, $w_{2}$ represents the rank summation of 100 Gy, and the test hypotheses are $H_{0}:\mu_{1}=\mu_{2}$ and $H_{1}:\mu_{1}<\mu_{2}$. The test statistics are the minimum of $\mu_{1}$ and $\mu_{2},$ where $u_{1}=w_{1}-\frac{n_{1}\left(n_{1}+1\right)}{2}=19-\frac{5*6}{2}=4$, and $u_{2}=w_{2}-\frac{n_{2}\left(n_{2}+1\right)}{2}=72-\frac{8*9}{2}\;$= 36. The significance value α was designated as 0.05. According to the table of the Wilcoxon rank-sum test, the critical region was 8. Since $\mu_{1}=4,$ which is less than 8, the null hypothesis was rejected, and the analysis indicated that the success rate of parthenogenetic experiments for 130+ Gy was significantly smaller than that of 100 Gy.

## 4. Conclusions

Gamma-induced parthenogenesis has shown some success in obtaining DH plants; however, this method has not been routinely applied due to many technological constraints. In this study, we have provided additional information, including a modified irradiation protocol, male pollinators with an identifiable phenotype in the hybrid offspring, and SSR markers, which has improved the efficiency in the identification and development of DH plants. Our results provide the possibility of obtaining a few DH sunflower plants using 2–4 flower heads per experiment and indicated that both male and female genotypes were responsible for the variations in the parthenogenetic response. In addition, this method needs preliminary experiments to determine the optimal doses of gamma rays for new sunflower genotypes. Since self-pollination can occur when CMS or emasculated sunflower lines are used as female plants, great care is required to avoid using sunflower heads with self-fertile disk florets. Lastly, although gamma-induced parthenogenesis can generate DH lines in a relatively straightforward manner, the current method cannot be used to perform large-scale production of DH plants; thus, there is still room for improvement in the existing technology.

## Figures and Tables

**Table 1:** Results of embryo rescue and progeny evaluation for CMS crosses. Progeny evaluation was performed based on hypocotyl color, flower color, fertility, and molecular marker (SSR) testing.

Cross	Gy	Pollination Date	Seed #/ Head #	Total # of Embroys Formed	Total # of Survived Plants	Survival Rate (%)	Total # of Plants with Green Hypocotyl	Percentage of Seedlings with Green Hypocotyl (%)	Offspring with Green Hypocotyl and Sterile Yellow Flowers	Total # of Plants with Female Parental Genotype	Percentage of Parthenogenesis (%)
291 × 1440	50	January 2017	738/6	508	170	33.46	5	2.94	2	2	1.18
291 × 1485	50	June 2018	287/4	161	161	100.00	4	2.48	2	2	1.24
303 × 1440	50	January 2017	1273/7	782	220	28.13	10	4.55	4	4	1.82
303 × 1440	50	October 2017	805/2	606	606	100.00	12	1.98	4	5	0.83
303 × 1440	50	February 2018	284/2	214	214	100.00	7	3.27	2	2	0.93
303 × 1440	50	June 2018	480/4	370	370	100.00	4	1.08	0	1	0.27
291 × 1440	100	October 2017	133/2	47	47	100.00	2	4.26	1	1	2.13
291 × 1440	100	June 2018	284/4	82	82	100.00	3	3.66	1	1	1.22
291 × 1440	100	October 2019	17/2	11	8	72.73	2	25.00	2	2	25.00
291 × 1485	100	February 2018	47/2	21	21	100.00	2	9.52	1	1	4.76
291 × 1485	100	June 2018	211/4	64	64	100.00	2	3.13	1	1	1.56
291 × 1485	100	October 2019	77/2	28	7	25.00	1	14.29	1	1	14.29
303 × 1440	100	February 2018	135/2	74	74	100.00	3	4.05	1	1	1.35
303 × 1440	100	June 2018	331/4	200	200	100.00	3	1.50	2	2	1.00
303 × 1485	100	February 2018	152/1	41	41	100.00	3	7.32	2	2	4.88
303 × 1485	100	June 2018	100/4	45	45	100.00	5	11.11	2	2	4.44
291 × 1440	130	February 2018	2312	8	8	100.00	1	12.50	1	1	12.50
303 × 1485	130	June 2018	78/4	26	26	100.00	2	7.69	1	1	3.85
303 × 1440	150	Juie 2018	47/4	20	20	100.00	5	25.00	3	4	20.00
303 × 1440	170	October 2017	165/2	9	9	100.00	1	11.11	1	1	11.11
291 × 1440	200	June 2018	27/4	22	22	100.00	5	22.73	2	4	18.18
303 × 1440	200	June 2018	14/4	7	7	100.00	1	14.29	1	1	14.29

**Table 2:** Results of embryo rescue and progeny evaluation for ENMS crosses. Progeny evaluation was performed based on hypocotyl color and molecular marker (SSR) testing.

Cross	Gy	Pollination Date	Seed #/Head #	Total # of Embroys Formed	Total # of Survived Plants	Survival Rate (%)	Total # of Plants with Green Hypocotyl	Percentage of Seedlings with Green Hypocotyl (%)	Offspring with Green Hypocotyl and Sterile Yellow Flowers	Total # of Plants with Female Parental Genotype	Percentage of Parthenogenesis (%)
CMS 291 × ANN1811	100	February 2019	21/2	14	6	42.86	NA	NA	1	1	16.67
H6. 60 × 1440	100	June 2019	9/2	6	6	100.00	1	16.67		1	16.67
HA 60 × 1440	100	October 2019	8/2	6	6	100.00	1	16.67		1	16.67
HA 60 × ANN1811	100	June 2019	11/2	6	6	100.00	1	16.67		1	16.67
HA 60 × ANN1811	100	October 2019	26/2	23	23	100.00	3	13.04		2	8.70
HA 89 × 1440	100	October 2019	26/2	16	16	100.00	5	31.25		1	6.25
HA 89 × ANN1811	100	June 2019	12/2	10	10	100.00	3	30.00		2	20.00
HA 89 × ANN1811	100	April 2020	35/6	23	23	100.00	4	17.39		4	17.39
HA 60 × 1440	130	October 2019	15/2	12	12	100.00	3	25.00		1	8.33
HA 60 × ANN1811	150	Aril 2020	11/4	9	9	100.00	2	22.22		2	22.22
HA 89 × ANN1811	150	April 2020	20/6	19	19	100.00	6	31.58		5	26.32
HA 60 × ANN1811	200	April 2020	8/4	4	4	100.00	1	25.00		1	25.00
HA 89 × ANN1811	200	April 2020	13/6	11	11	100.00	5	45.45		5	45.45

**Table 3:** All DH offspring obtained from the plant materials used in this project.

Female	Pollen Source	Gamma Ray Dose	Obtained DH Offspring
CMS 291	G11/1440	50 Gy	2
		100 Gy	4
		130 Gy	1
		200 Gy	4
CMS 291	G11/1485	50 Gy	2
		100 Gy	3
CMS 291	ANN1811	100 Gy	1
CMS 303	G11/1440	50 Gy	12
		100 Gy	3
		150 Gy	4
		170 Gy	1
		200 Gy	1
CMS 303	G11/1485	100 Gy	4
		130 Gy	1
HA 60	G11/1440	100 Gy	2
		130 Gy	1
HA 60	ANN1811	100 Gy	3
		150 Gy	2
		200 Gy	1
HA 89	G11/1440	100 Gy	1
HA 89	ANN1811	100 Gy	6
		150 Gy	5
		200 Gy	5
Total			69

## Data Availability

Data is contained within the article or Supplementary Material.

## Supplementary Materials

The following supporting information can be downloaded at: https://www.mdpi.com/article/10.3390/plants12132430/s1. Supplemental Figure S1. Molecular marker evaluation. Supplemental Figure S2. Chromosome counting. Supplemental Figure S3. Determination of emasculation conditions. Supplemental Table S1. Progeny evaluation for cytoplasmic male sterility (CMS) and emasculated non-male sterile (ENMS) sunflower crosses. Supplemental Table S2. Simple sequence repeat (SSR) primers. Supplemental Table S3. Statistical analyses. Supplemental Table S4. Summary of germination rate, chromosome number, and pollination rate for the wild-type sunflower germplasm.

## References

[1] Germanà M.A. (2011). Anther culture for haploid and doubled haploid production. Plant Cell Tissue Organ. Cult..

[2] Prigge V., Xu X., Li L., Babu R., Chen S., Atlin G.N., Melchinger A.E. (2012). New insights into the genetics of in vivo induction of maternal haploids, the backbone of doubled haploid technology in maize. Genetics.

[3] Dhooghe E., Van Laere K., Eeckhaut T., Leus L., Van Huylenbroeck J. (2011). Mitotic chromosome doubling of plant tissues in vitro. Plant Cell Tissue Organ. Cult..

[4] Manzoor A., Ahmad T., Bashir M.A., Hafiz I.A., Silvestri C. (2019). Studies on colchicine induced chromosome doubling for enhancement of quality traits in ornamental plants. Plants.

[5] Weyen J., Segui-Simarro J.M. (2021). Applications of doubled haploids in plant breeding and applied research. Doubled Haploid Technology, Methods in Molecular Biology.

[6] Ren J., Wu P., Trampe B., Tian X., Lübberstedt T., Chen S. (2017). Novel technologies in doubled haploid line development. Plant Biotechnol. J..

[7] Niazian M., Shariatpanahi M.E. (2020). In Vitro-based doubled haploid production: Recent improvements. Euphytica.

[8] Kalinowska K., Chamas S., Unkel K., Demidov D., Lermontova I., Dresselhaus T., Kumlehn J., Dunemann F., Houben A. (2019). State-of-the-art and novel developments of in vivo haploid technologies. Theor. Appl. Genet..

[9] Zhang Y.X., Lespinasse Y. (1991). Pollination with gamma-irradiated pollen and development of fruits, seeds and parthenogenetic plants in apple. Euphytica.

[10] Falque M., Kodia A., Sounigo O., Eskes A., Charrier A. (1992). Gamma-irradiation of cacao (*Theobroma cacao* L.) pollen: Effect on pollen grain viability, germination and mitosis and on fruit set. Euphytica.

[11] Caglar G., Abak K. (1999). Progress in the production of haploid embryos, plants and doubled haploids in cucumber (*C. sativus* L.) by gamma irradiated pollen, in Turkey. Acta Hortic..

[12] Taşkın H., Yücel N.K., Baktemur G., Çömlekçioğlu S., Büyükalaca S. (2013). Effects of different genotypes and gamma ray doses on haploidization with irradiated pollen technique in watermelon (*Citrullus lanatus* L.). Can. J. Plant Sci..

[13] Bagheri L., Lotfi M., Nori M., Sivasankar S., Ellis N., Jankuloski L., Ingelbrecht I. (2021). Production of Haploid Embryos and Plants in Iranian Melon (*Cucumis melo* L.) through Irradiated Pollen-Induced Parthenogenesis. Mutation Breeding, Genetic Diversity and Crop Adaptation to Climate Change.

[14] Blinkov A.O., Varlamova N.V., Kurenina L.V., Khaliluev M.R. (2022). The production of Helianthus haploids: A review of its current status and future prospects. Plants.

[15] Gürel A., Kontowski S., Nichterlein K., Friedt W. (1991). Embryogenesis in microspore culture of sunflower (*Helianthus annuus* L.). Helia.

[16] Todorova M., Dalhoff M., Friedt W. (1993). Microspore culture in sunflower (*Helianthus annuus* L.). Biotechnol. Biotechnol. Equip..

[17] Coumans M., Zhong D. (1995). Doubled haploid sunflower (*Helianthus annuus*) plant production by androgenesis: Fact or artifact? Part 2. In vitro isolated microspore culture. Plant Cell Tissue Org. Cult..

[18] Todorova M., Ivanov P., Shindrova P., Christov M., Ivanova I. (1997). Doubled haploid production of sunflower (*Helianthus annuus* L.) through irradiated pollen-induced parthenogenesis. Euphytica.

[19] Todorova M., Ivanov P., Nenova N., Encheva J. (2004). Effect of female genotype on the efficiency of γ-induced parthenogenesis in sunflower (*Helianthus annuus*). Helia.

[20] Drumeva M., Berville A., Ivanov P., Nenova N., Encheva J. (2005). Molecular investigations on the doubled haploid origin of sunflower lines (*Helianthus annuus* L.) developed through gamma-induced parthenogenesis. Biotechnol. Biotechnol. Equip..

[21] Drumeva M., Yankov P. (2015). Investigation on the parthenogenetic response of sunflower lines and hybrids. Agric. Sci. Technol..

[22] Guichoux E., Lagache L., Wagner S., Chaumeil P., Léger P., Lepais O., Lepoittevin C., Malausa T., Revardel E., Salin F. (2011). Current trends in microsatellite genotyping. Mol. Ecol. Resour..

[23] Testillano P., Georgiev S., Mogensen H.L., Coronado M.J., Dumas C., Risueno M.C., Matthys-Rochon E. (2004). Spontaneous chromosome doubling results from nuclear fusion during in vitro maize induced microspore embryogenesis. Chromosoma.

[24] Coe E.H. (1959). A line of maize with high haploid frequency. Am. Nat..

[25] Jacquier N.M.A., Gilles L.M., Martinant J.P., Rogowsky P.M., Widiez T. (2021). Maize in planta haploid inducer lines: A cornerstone for doubled haploid technology. Methods Mol. Biol..

[26] Pandey K.K., Phung M. (1982). Hertwig effect in plants: Induced parthenogenesis through the use of irradiated pollen. Theor. Appl. Genet..

[27] Sukno S., Ruso J., Jan C.C., Melero-Vara J.M., Fernández-martínez J.M. (1999). Interspecific hybridization between sunflower and wild perennial Helianthus species via embryo rescue. Euphytica.

[28] Liu Z., Mulpuri S., Feng J., Vick B.A., Jan C.C. (2012). Molecular mapping of the Rf_3_ fertility restoration gene to facilitate its utilization in breeding confection sunflower. Mol. Breed..

[29] Qi L., Gulya T., Seiler G.J., Hulke B.S., Vick B.A. (2011). Identification of resistance to new virulent races of rust in sunflowers and validation of DNA markers in the gene pool. Phytopathology.

[30] Jan C.C. (1996). Developing unique interspecific germplasm for sunflower improvement. Biotechnology & wild species. Proceedings of the 14th International Sunflower Conference.

