# Supplementary material for: Irradiated Pollen-Induced Parthenogenesis for Doubled Haploid Production in Sunflowers (Helianthus spp.)
Source: Plants (Basel). 2023 Jun 23;12(13):2430. doi: 10.3390/plants12132430 (PMC10346741; doi:10.3390/plants12132430)
Supplement: Supplementary file 1 [file plants-12-02430-s001.zip › Supplemental Figure S1.pptx]

## Slide 1
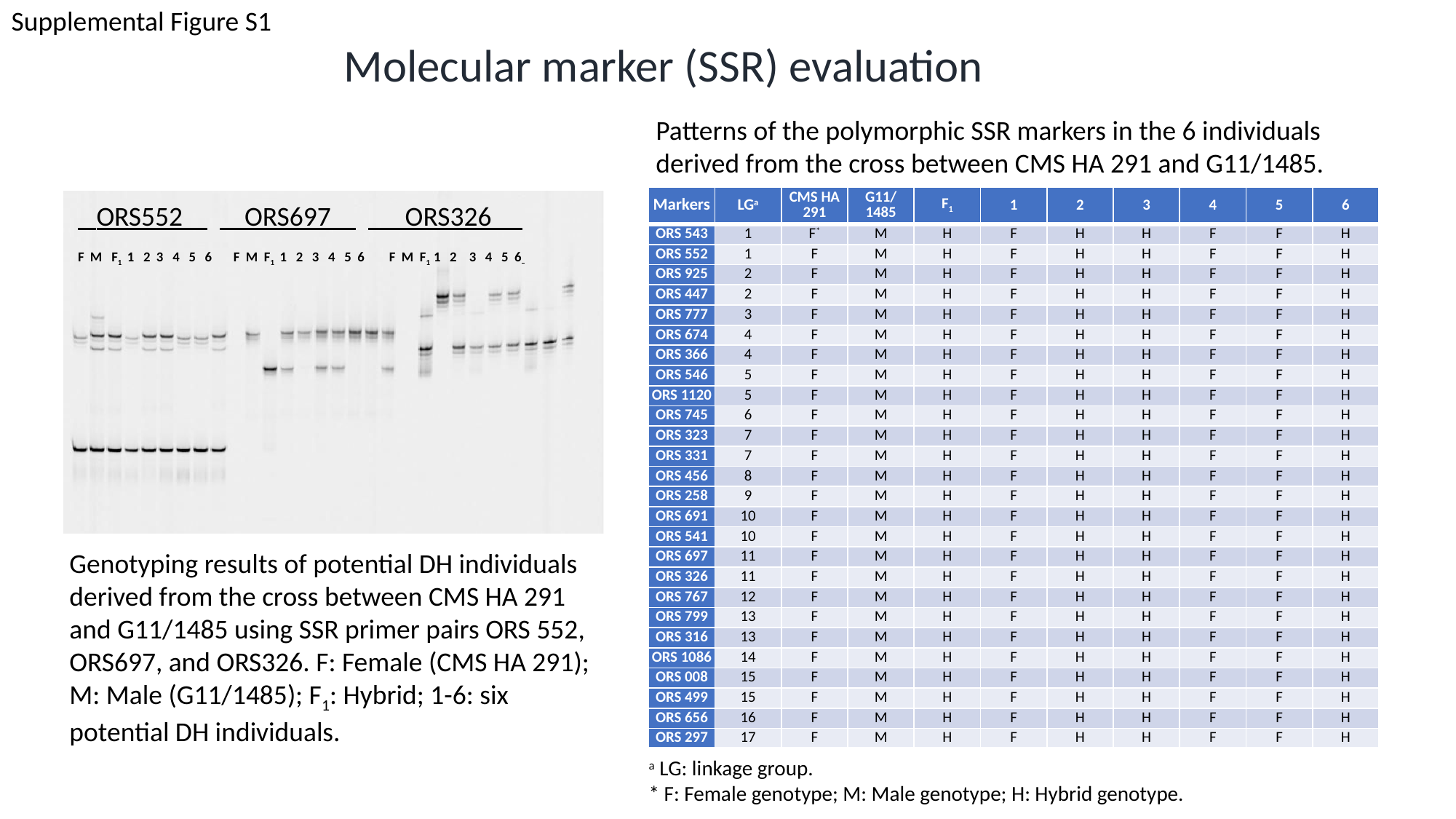

Supplemental Figure S1
Molecular marker (SSR) evaluation
Patterns of the polymorphic SSR markers in the 6 individuals derived from the cross between CMS HA 291 and G11/1485.
| Markers | LGa | CMS HA 291 | G11/ 1485 | F1 | 1 | 2 | 3 | 4 | 5 | 6 |
| --- | --- | --- | --- | --- | --- | --- | --- | --- | --- | --- |
| ORS 543 | 1 | F\* | M | H | F | H | H | F | F | H |
| ORS 552 | 1 | F | M | H | F | H | H | F | F | H |
| ORS 925 | 2 | F | M | H | F | H | H | F | F | H |
| ORS 447 | 2 | F | M | H | F | H | H | F | F | H |
| ORS 777 | 3 | F | M | H | F | H | H | F | F | H |
| ORS 674 | 4 | F | M | H | F | H | H | F | F | H |
| ORS 366 | 4 | F | M | H | F | H | H | F | F | H |
| ORS 546 | 5 | F | M | H | F | H | H | F | F | H |
| ORS 1120 | 5 | F | M | H | F | H | H | F | F | H |
| ORS 745 | 6 | F | M | H | F | H | H | F | F | H |
| ORS 323 | 7 | F | M | H | F | H | H | F | F | H |
| ORS 331 | 7 | F | M | H | F | H | H | F | F | H |
| ORS 456 | 8 | F | M | H | F | H | H | F | F | H |
| ORS 258 | 9 | F | M | H | F | H | H | F | F | H |
| ORS 691 | 10 | F | M | H | F | H | H | F | F | H |
| ORS 541 | 10 | F | M | H | F | H | H | F | F | H |
| ORS 697 | 11 | F | M | H | F | H | H | F | F | H |
| ORS 326 | 11 | F | M | H | F | H | H | F | F | H |
| ORS 767 | 12 | F | M | H | F | H | H | F | F | H |
| ORS 799 | 13 | F | M | H | F | H | H | F | F | H |
| ORS 316 | 13 | F | M | H | F | H | H | F | F | H |
| ORS 1086 | 14 | F | M | H | F | H | H | F | F | H |
| ORS 008 | 15 | F | M | H | F | H | H | F | F | H |
| ORS 499 | 15 | F | M | H | F | H | H | F | F | H |
| ORS 656 | 16 | F | M | H | F | H | H | F | F | H |
| ORS 297 | 17 | F | M | H | F | H | H | F | F | H |
 ORS552 ORS697 ORS326
F M F1 1 2 3 4 5 6 F M F1 1 2 3 4 5 6 F M F1 1 2 3 4 5 6
Genotyping results of potential DH individuals derived from the cross between CMS HA 291 and G11/1485 using SSR primer pairs ORS 552, ORS697, and ORS326. F: Female (CMS HA 291); M: Male (G11/1485); F1: Hybrid; 1-6: six potential DH individuals.
a LG: linkage group.
* F: Female genotype; M: Male genotype; H: Hybrid genotype.
