# Supplementary material for: Irradiated Pollen-Induced Parthenogenesis for Doubled Haploid Production in Sunflowers (Helianthus spp.)
Source: Plants (Basel). 2023 Jun 23;12(13):2430. doi: 10.3390/plants12132430 (PMC10346741; doi:10.3390/plants12132430)
Supplement: Supplementary file 1 [file plants-12-02430-s001.zip › Supplemental Figure S2.pptx]

## Slide 1
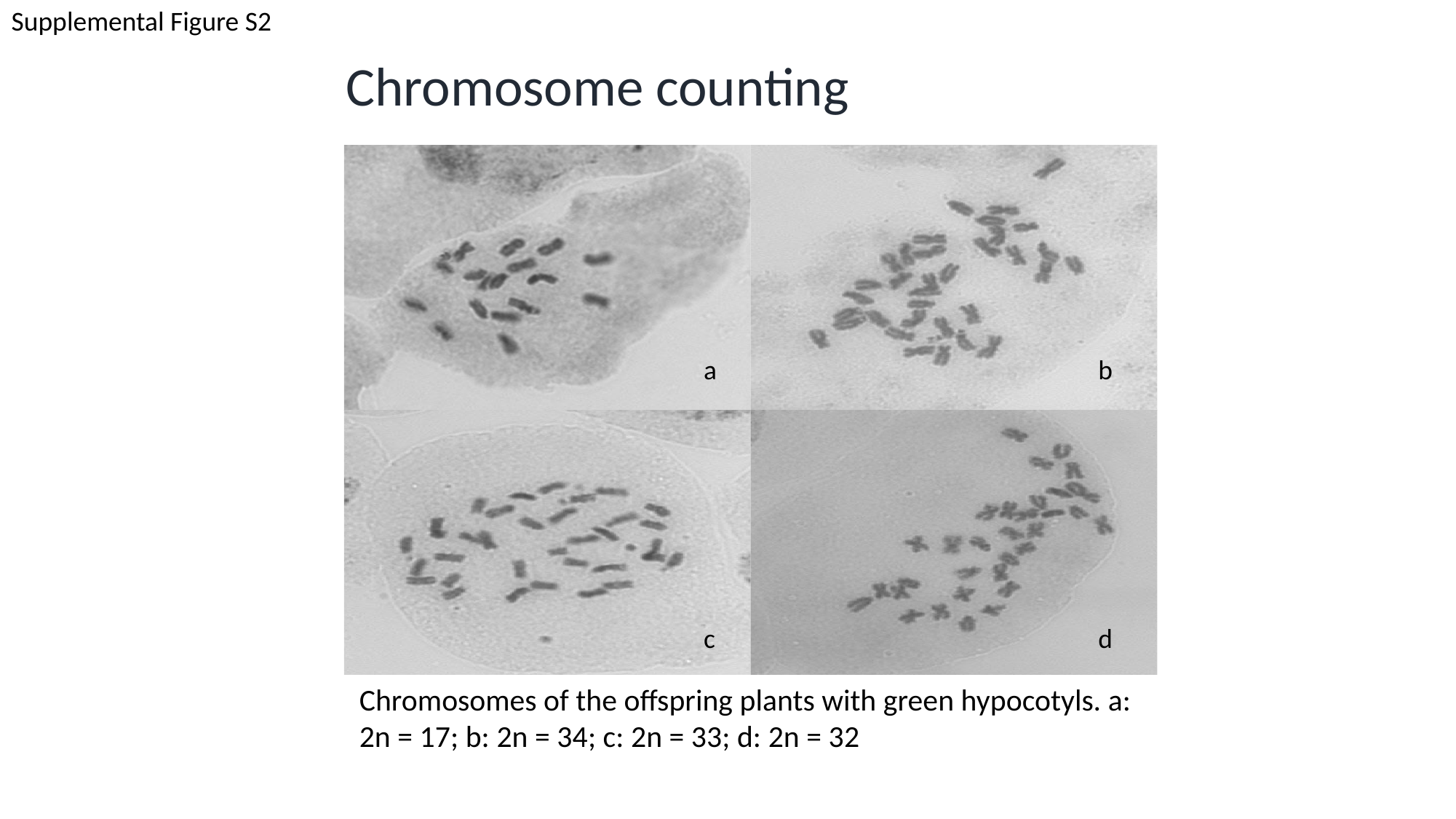

Supplemental Figure S2
Chromosome counting
a
b
c
d
Chromosomes of the offspring plants with green hypocotyls. a: 2n = 17; b: 2n = 34; c: 2n = 33; d: 2n = 32
