# Supplementary material for: Irradiated Pollen-Induced Parthenogenesis for Doubled Haploid Production in Sunflowers (Helianthus spp.)
Source: Plants (Basel). 2023 Jun 23;12(13):2430. doi: 10.3390/plants12132430 (PMC10346741; doi:10.3390/plants12132430)
Supplement: Supplementary file 1 [file plants-12-02430-s001.zip › Supplemental Figure S3.pptx]

## Slide 1
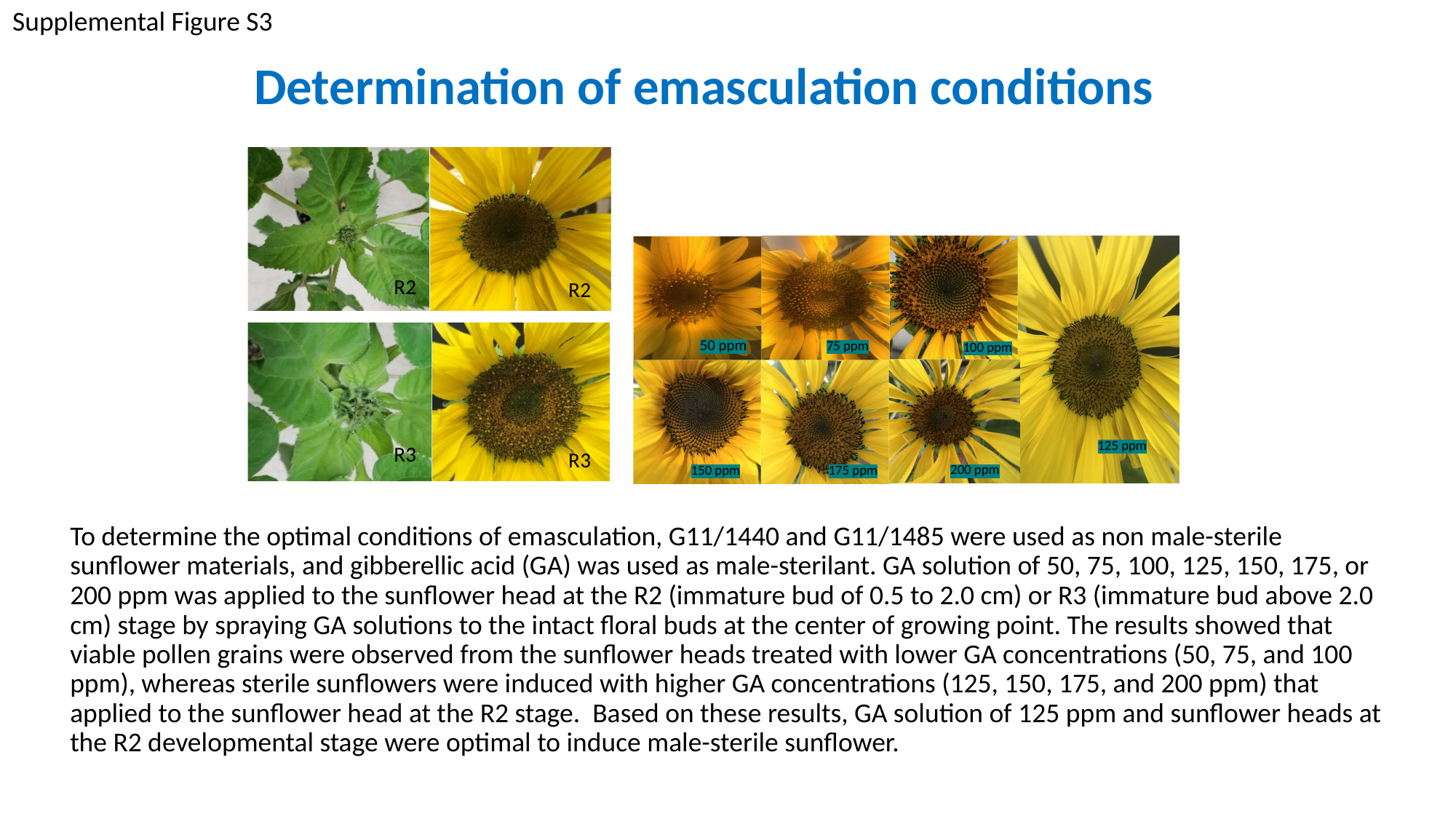

Supplemental Figure S3
Determination of emasculation conditions
R2
R2
R3
R3
100 ppm
125 ppm
75 ppm
50 ppm
200 ppm
150 ppm
175 ppm
To determine the optimal conditions of emasculation, G11/1440 and G11/1485 were used as non male-sterile sunflower materials, and gibberellic acid (GA) was used as male-sterilant. GA solution of 50, 75, 100, 125, 150, 175, or 200 ppm was applied to the sunflower head at the R2 (immature bud of 0.5 to 2.0 cm) or R3 (immature bud above 2.0 cm) stage by spraying GA solutions to the intact floral buds at the center of growing point. The results showed that viable pollen grains were observed from the sunflower heads treated with lower GA concentrations (50, 75, and 100 ppm), whereas sterile sunflowers were induced with higher GA concentrations (125, 150, 175, and 200 ppm) that applied to the sunflower head at the R2 stage. Based on these results, GA solution of 125 ppm and sunflower heads at the R2 developmental stage were optimal to induce male-sterile sunflower.
