# Supplementary material for: Irradiated Pollen-Induced Parthenogenesis for Doubled Haploid Production in Sunflowers (Helianthus spp.)
Source: Plants (Basel). 2023 Jun 23;12(13):2430. doi: 10.3390/plants12132430 (PMC10346741; doi:10.3390/plants12132430)
Supplement: Supplementary file 1 [file plants-12-02430-s001.zip › Supplemental Table S3.pdf]

Supplemental Table S3. Statistical analyses

1. The mean percentages of parthenogenesis were positively correlated with the increase of gamma ray doses

A. Gamma ray doses affected the mean percentages of parthenogenesis for CMS sunflower crosses.

Using CMS sunflower lines as female parents, results were obtained from 6 Gy irradiation types (50 Gy, 100 Gy, 130 Gy, 150 Gy, 170 Gy, and 200 Gy). The Kruskal-Wallis test was used to analyze the equality of means (mean percentages of parthenogenesis) when the assumption is that the samples were not guaranteed from populations with normal distribution. The sample sizes of 6 different Gy irradiation types are listed in the following table:

Gy type	Sample Size
50 Gy	6
100 Gy	10
130 Gy	2
150 Gy	1
170 Gy	1
200 Gy	2

The sample sizes of 130 Gy, 150 Gy, 170 Gy, and 200 Gy types were all below 5. Since Kruskal-Wallis test performs well when the sample size is greater or equals to 5, we combined the data of 130 Gy, 150 Gy, 170 Gy, and 200 Gy into one group and named this sample type as 130+ Gy. After regrouping, we had the following three sample sizes:

Gy type	Sample Size
50 Gy	n_1= 6
100 Gy	n_2=10
130+ Gy	$n_3 = 6$

Notation:

μ_1 = the mean percentage of parthenogenesis of 50 Gy

μ_2 = the mean percentage of parthenogenesis of 100 Gy

μ_3 = the mean percentage of parthenogenesis of 130+ Gy.

k = the number of population ($k = 3$ for our case)

n_i = the size of sample i (n_1= 6, n_2=10, $n_3 = 6$)

n_T= total number of observations in all samples ($n_T = 6 + 10 + 6 = 22$)

R_i = the sum of the ranks for sample i ($R_1 = 28, R_2 = 121, R_3 = 104$)

Gy	Percentage of parthenogenesis (%)	Rank	Summation of rank of same Gy type
50	0.27	1	$R_1 = 28$
50	0.83	2	
50	0.94	3	
50	1.18	5	
50	1.24	7	
50	1.82	10	
100	1.00	4	$R_2 = 121$
100	1.22	6	

100	1.35	8	
100	1.56	9	
100	2.13	11	
100	4.44	13	
100	4.76	14	
100	4.88	15	
100	14.29	19	
100	25.00	22	
130	3.85	12	$R_3 = 104$
130	12.50	17	
150	20.00	21	
170	11.11	16	
200	14.29	18	
200	18.18	20	

The test hypotheses are:

$H_0: \mu_1 = \mu_2 = \mu_3$ [the 3 group (50 Gy, 100 Gy, and 130+ Gy) had the same mean]
$H_{1:}$ The three means were not all equal

The test statistics is:

$$H = \left[\frac{12}{n_T(n_T+1)} \sum_{i=1}^{k} \frac{R_i^2}{n_i}\right] - 3(n_T + 1) = \left[\frac{12}{22(22+1)} \left(\frac{28^2}{6} + \frac{121^2}{10} + \frac{104^2}{6}\right)\right] - 3(22 + 1) = 11.572$$

The test statistic follows a χ^2 distribution with degree of freedom 2.
The p value of the test is $p(\chi^2 > 11.572) = .0031$
The chosen significance value of the test was 0.05. Since .0031 < 0.05, we rejected the null hypothesis H_0, and concluded that the mean percentages of parthenogenesis for three Gy types (50 Gy, 100 Gy, and 130+ Gy) were significantly different.

B. Gamma ray doses affected the mean percentages of parthenogenesis for emasculated non-male sterile (ENMS) sunflower crosses.

For studies using ENMS sunflower lines as female parents (including one data obtained from CMS 291 x ANN1811), results were obtained from four Gy irradiation types (100 Gy, 130 Gy, 150 Gy, and 200 Gy). The date set is as following:

Gy	Sample size
100	8
130	1
150	2
200	2

Since the sample size for the 130 Gy, 150 Gy, and 200 Gy groups were small, we combined them into one group and named it 130+ Gy. After regrouping, the data set is as following:

Gy	Sample size
130+	$n_1 = 5$
100	$n_2 = 8$

Wilcoxon two-sample test was used to determine if 130+ Gy produced higher mean percentage of parthenogenesis.

In this analysis:

n_1= the sample size of 130+ Gy = 5
n_2 = the sample size of 100 Gy = 8
μ_1 = mean percentage of parthenogenesis of 130+ Gy
μ_2 = mean percentage of parthenogenesis of 100 Gy
w_1 represents the rank summation of 130+ Gy.
w_2 represents the rank summation of 100 Gy.

The hypothesis is set up as
$H_0: \mu_1 = \mu_2$
$H_1: \mu_1 > \mu_2$

Gy	Percentage of parthenogenesis (%)	Rank	Summation of rank of same Gy type
130	8.33	2	$w_1 = 48$
150	22.22	10	
150	26.32	12	
200	25	11	
200	45.45	13	
100	6.25	1	$w_2 = 43$
100	8.7	3	
100	16.67	4	
100	16.67	5	
100	16.67	6	
100	16.67	7	
100	17.39	8	
100	20	9	

The test statistic is the minimum of μ_1 and μ_2, where

$$u_1 = w_1 - \frac{n_1(n_1+1)}{2} = 48 - \frac{5*6}{2} = 33$$

$$u_2 = w_2 - \frac{n_2(n_2+1)}{2} = 43 - \frac{8*9}{2} = 7$$

Table A.17 (continued) Critical Values for the Wilcoxon Rank-Sum Test

One-Tailed Test at $\alpha = 0.025$ or Two-Tailed Test at $\alpha = 0.05$

	n_2																
n_1	**4**	**5**	**6**	**7**	**8**	**9**	**10**	**11**	**12**	**13**	**14**	**15**	**16**	**17**	**18**	**19**	**20**
**1**																	
**2**					0	0	0	0	1	1	1	1	1	2	2	2	2
**3**		0	1	1	2	2	3	3	4	4	5	5	6	6	7	7	8
**4**	0	1	2	3	4	4	5	6	7	8	9	10	11	11	12	13	13
**5**		2	3	5	6	7	8	9	11	12	13	14	15	17	18	19	20
**6**			5	6	8	10	11	13	14	16	17	19	21	22	24	25	27
**7**				8	10	12	14	16	18	20	22	24	26	28	30	32	34
**8**					13	15	17	19	22	24	26	29	31	34	36	38	41
**9**						17	20	23	26	28	31	34	37	39	42	45	48
**10**							23	26	29	33	36	39	42	45	48	52	55
**11**								30	33	37	40	44	47	51	55	58	62
**12**									37	41	45	49	53	57	61	65	69
**13**										45	50	54	59	63	67	72	76
**14**											55	59	64	67	74	78	83
**15**												64	70	75	80	85	90
**16**													75	81	86	92	98
**17**														87	93	99	105
**18**															99	106	112
**19**																113	119
**20**																	127

One-Tailed Test at $\alpha = 0.05$ or Two-Tailed Test at $\alpha = 0.1$

	n_2																	
n_1	**3**	**4**	**5**	**6**	**7**	**8**	**9**	**10**	**11**	**12**	**13**	**14**	**15**	**16**	**17**	**18**	**19**	**20**
**1**																	0	0
**2**			0	0	0	1	1	1	1	2	2	3	3	3	3	4	4	4
**3**	0	0	1	2	2	3	4	4	5	5	6	7	7	8	9	9	10	11
**4**		1	2	3	4	5	6	7	8	9	10	11	12	14	15	16	17	18
**5**			4	5	6	8	9	11	12	13	15	16	18	19	20	22	23	25
**6**				7	8	10	12	14	16	17	19	21	23	25	26	28	30	32
**7**					11	13	15	17	19	21	24	26	28	30	33	35	37	39
**8**						15	18	20	23	26	28	31	33	36	39	41	44	47
**9**							21	24	27	30	33	36	39	42	45	48	51	54
**10**								27	31	34	37	41	44	48	51	55	58	62
**11**									34	38	42	46	50	54	57	61	65	69
**12**										42	47	51	55	60	64	68	72	77
**13**											51	56	61	65	70	75	80	84
**14**												61	66	71	77	82	87	92
**15**													72	77	83	88	94	100
**16**														83	89	95	101	107
**17**															96	102	109	115
**18**																109	116	123
**19**																	123	130
**20**																		138

The chosen significance value of the test was $\alpha = 0.05$. According to the table of the Wilcoxon Rank-Sum test (see above, One-Tailed Test at α = 0.05), the critical region *is* 8. Since $\mu_2 = 7 < 8$, we rejected the null hypothesis and concluded that the mean percentage of parthenogenesis for 130+ Gy was significantly higher than that of 100 Gy.

C. Figure 4:

The values for the mean percentage and standard errors of different Gy types for CMS crosses (CMS mean and CMS se) and ENMS crosses (ENMS mean and ENMS se) can be found in Supplemental Table S1, spreadsheet "CMS progeny evaluation" and spreadsheet "ENMS progeny evaluation" (highlighted in yellow).

	CMS mean	ENMS mean	CMS se	ENMS se
50 Gy	1.04		0.21	
100 Gy	6.06	14.88	2.45	1.68
130+ Gy	13.32	25.47	2.34	5.94

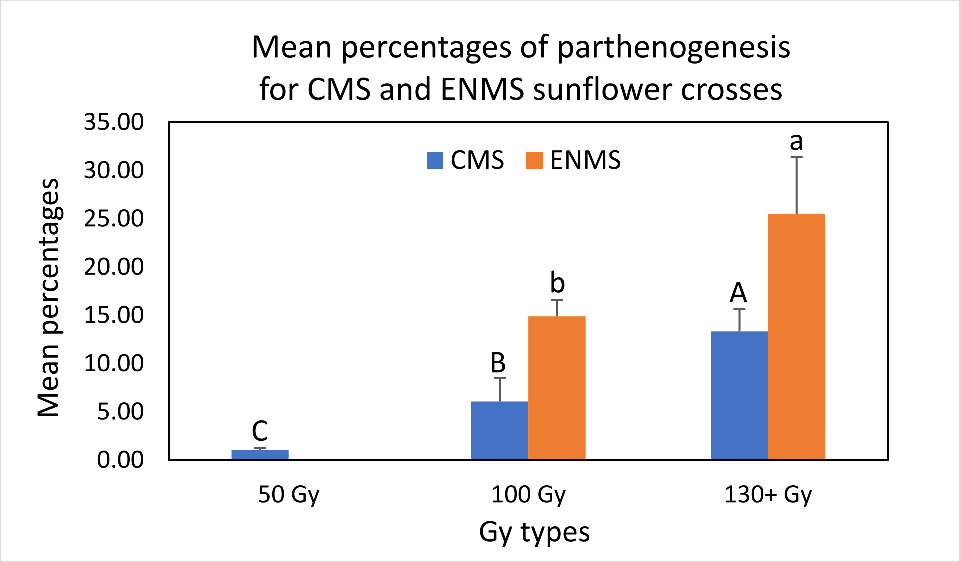

Figure 4. The mean percentages of parthenogenesis of different gamma ray dose (Gy) types. The Kruskal-Wallis test was used to analyze the equality of the mean percentages of parthenogenesis for cytoplasmic male sterility (CMS) sunflower crosses; in this analysis, 130+ Gy is the combined data of 130 Gy, 150 Gy, 170 Gy, and 200 Gy. The Wilcoxon two-sample test was used to analyze the equality of the mean percentages of parthenogenesis for emasculated non-male sterile (ENMS) sunflower crosses; in this analysis, 130+ Gy is the combined data of 130 Gy, 150 Gy, and 200 Gy. Error bars show range of the mean percentages of parthenogenesis. Mean percentages labeled by the different letter are significantly dissimilar ($P < 0.05$).

2. Gamma ray dose at 100 Gy generated the highest success rate of the parthenogenetic experiments

A. Gamma ray doses affected the overall success rates of parthenogenetic experiments for CMS sunflower crosses.

The analysis in the first part shows that gamma ray doses affected the mean percentages of parthenogenesis for both CMS and ENMS sunflower crosses. Specifically, as gamma ray doses increased from 50 Gy to 100 Gy, then to 130+ Gy, the mean percentages of parthenogenesis increased as well (1.04%, 6.06%, and 13.32%, respectively). However, the data also showed that gamma ray doses affected the overall success rate of parthenogenesis experiments. In this analysis, 16 separate experiments (4 crosses x 4 replicates per cross) were performed for each of the six gamma ray doses (50 Gy, 100 Gy, 130 Gy, 150 Gy, 170 Gy, and 200 Gy). The numbers of parthenogenetic plants and success rates of the experiments are shown in the table below:

Gy-	Total number of experiments per gamma ray dose	Number of parthenogenetic plants obtained	Success rate of the experiments
50 Gy	16	6	0.38
100 Gy	16	10	0.63
130 Gy	16	2	0.13
150 Gy	16	1	0.06
170 Gy	16	1	0.06
200 Gy	16	2	0.13

In order to choose the most appropriate gamma ray dose, we not only need to consider the effects of gamma ray doses on the mean percentages of parthenogenesis, but also should consider the effects of gamma ray doses on the overall success rate of parthenogenetic experiments. The percentages of parthenogenesis were therefore modified by multiplying a coefficient of the overall success rate of the experiments. The results are shown in the following table:

Gy	Percentage of parthenogenesis (%)	Success rate of the experiments	Modified Percentage of parthenogenesis (%)	Rank	Rank summation of same Gy type
50	0.27	0.38	0.10	1	$R_1 = 24$
50	0.83	0.38	0.31	2	
50	0.94	0.38	0.36	3	
50	1.18	0.38	0.45	4	
50	1.24	0.38	0.47	5	
50	1.82	0.38	0.69	9	
100	1.00	0.63	0.63	7	$R_2 = 154$
100	1.22	0.63	0.77	10	
100	1.35	0.63	0.85	11	
100	1.56	0.63	0.98	12	
100	2.13	0.63	1.34	14	
100	4.44	0.63	2.80	18	
100	4.76	0.63	3.00	19	
100	4.88	0.63	3.07	20	
100	14.29	0.63	9.00	21	
100	25.00	0.63	15.75	22	
130	3.85	0.13	0.50	6	$R3 = 75$
130	12.50	0.13	1.63	15	
150	20.00	0.06	1.20	13	
170	11.11	0.06	0.67	8	
200	14.29	0.13	1.86	16	
200	18.18	0.13	2.36	17	

The Kruskal-Wallis test was used to analyze the equality of means (modified mean percentages of parthenogenesis) when the assumption is that the samples were not guaranteed from populations with normal distribution.

The test hypotheses are:

H_0: $\mu_1 = \mu_2 = \mu_3$ [the 3 group (50 Gy, 100 Gy, and 130+ Gy) had the same mean]
H_1: The three means were not all equal

The test statistics is:

$$H = \left[\frac{12}{n_T(n_T+1)} \sum_{i=1}^{k} \frac{R_i^2}{n_i}\right] - 3(n_T + 1) = \left[\frac{12}{22(22+1)} \left(\frac{24^2}{6} + \frac{154^2}{10} + \frac{75^2}{6}\right)\right] - 3(22 + 1) = 11.75$$

The test statistic follows a χ^2 distribution with degree of freedom 2.
The p value of the test is $p(\chi^2 > 11.75)$ = .0028
The chosen significance value of the test was 0.05. Since .0028 < .05, we rejected the null hypothesis H_0, and concluded that the mean percentages of parthenogenesis for three Gy types (50 Gy, 100 Gy, and 130+ Gy) were significantly different.

B. Gamma ray doses affected the overall success rates of parthenogenetic experiments for emasculated non-male sterile (ENMS) sunflower crosses.

The numbers of parthenogenetic plants and success rates of the experiments are shown in the table below:

Gy-	Total number of experiments per gamma ray dose	Number of parthenogenetic plants obtained	Success rate of the experiments
100 Gy	22	8	0.36
130 Gy	18	1	0.06
150 Gy	22	2	0.09
200 Gy	22	2	0.09

After modified the percentages of parthenogenesis by multiplying a coefficient of the overall success rate of the experiments, the results are shown in the following table:

Gy	Percentage of parthenogenesis (%)	Success rate of the experiments	Modified Percentage of parthenogenesis (%)	Rank	Rank summation of same Gy type
130	8.33	0.06	0.50	1	$w_1 = 19$
150	22.22	0.09	2.00	2	
150	26.32	0.09	2.37	5	
200	25	0.09	2.25	4	
200	45.45	0.09	4.09	7	
100	6.25	0.36	2.25	3	$w_2 = 72$
100	8.7	0.36	3.13	6	
100	16.67	0.36	6.00	8	
100	16.67	0.36	6.00	9	
100	16.67	0.36	6.00	10	
100	16.67	0.36	6.00	11	
100	17.39	0.36	6.26	12	
100	20	0.36	7.20	13	

Wilcoxon two-sample test was used to determine if 130+ Gy produced smaller modified mean percentage of parthenogenesis.

In this analysis:

n_1= the sample size of 130+ Gy = 5
n_2 = the sample size of 100 Gy = 8
μ_1 = mean percentage of parthenogenesis of 130+ Gy
μ_2 = mean percentage of parthenogenesis of 100 Gy
w_1 represents the rank summation of 130+ Gy.
w_2 represents the rank summation of 100 Gy.

The hypothesis is set up as
$H_0: \mu_1 = \mu_2$
$H_1: \mu_1 < \mu_2$

The test statistic is the minimum of μ_1 and μ_2, where

$$u_1 = w_1 - \frac{n_1(n_1 + 1)}{2} = 19 - \frac{5 * 6}{2} = 4$$

$$u_2 = w_2 - \frac{n_2(n_2+1)}{2} = 72 - \frac{8*9}{2} = 36$$

The chosen significance value of the test was $\alpha = 0.05$. According to the table of the Wilcoxon Rank-Sum test (see above, One-Tailed Test at α = 0.05), the critical region is 8. Since $\mu_1 = 4 < 8$, we rejected the null hypothesis and concluded that the mean percentage of parthenogenesis of 130+ Gy was significantly smaller than that of 100 Gy.

C. Figure 5

The values for the modified mean percentages and standard errors of different Gy types for CMS crosses (CMS mean and CMS se) and ENMS crosses (ENMS mean and ENMS se) can be found in Supplemental Table S1, spreadsheet "CMS progeny evaluation" and spreadsheet "ENMS progeny evaluation" (highlighted in yellow).

Gy	Modified CMS mean	Modified ENMS mean	CMS se	ENMS se
50 Gy	0.40		0.08	
100 Gy	3.82	5.36	1.54	0.60
130+ Gy	1.37	2.24	0.29	0.57

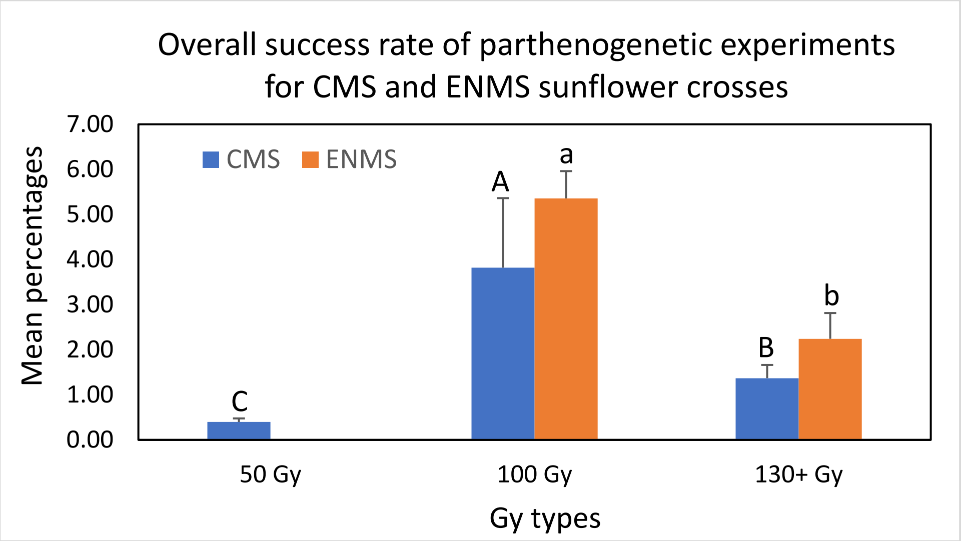

Figure 5. Overall success rate of parthenogenetic experiments for CMS and ENMS sunflower crosses. The Kruskal-Wallis test was used to analyze the overall success rate of parthenogenetic experiments for cytoplasmic male sterility (CMS) sunflower crosses; in this analysis, 130+ Gy is the combined data of 130 Gy, 150 Gy, 170 Gy, and 200 Gy. The Wilcoxon two-sample test was used to analyze the overall success rate of parthenogenetic experiments for emasculated non-male sterile (ENMS) sunflower crosses; in this analysis, 130+ Gy is the combined data of 130 Gy, 150 Gy, and 200 Gy. Error bars show range of the mean percentages of parthenogenesis. Mean percentages labeled by the different letter are significantly dissimilar ($P < 0.05$).
